# 15-lncRNA-Based Classifier-Clinicopathologic Nomogram Improves the Prediction of Recurrence in Patients with Hepatocellular Carcinoma

**DOI:** 10.1155/2020/9180732

**Published:** 2020-12-01

**Authors:** Qiong Zhang, Gang Ning, Hongye Jiang, Yanlin Huang, Jinsong Piao, Zhen Chen, Xiaojun Tan, Jiangyu Zhang, Genglong Liu

**Affiliations:** ^1^Department of Pathology, Affiliated Cancer Hospital & Institute of Guangzhou Medical University, Guangzhou, 510095 Guangdong Province, China; ^2^Department of Gastroenterology and Hepatology, Guangzhou Digestive Diseases Center, Guangzhou First People's Hospital, South China University of Technology, Guangzhou, 510180 Guangdong Province, China; ^3^Department of Clinical Laboratory, Shunde Hospital, Southern Medical University (The First People's Hospital of Shunde), Foshan, 528300 Guangdong Province, China; ^4^Department of Infectious Diseases, The Third Affiliated Hospital of Sun Yat-Sen University, Guangzhou, 510630 Guangdong Province, China

## Abstract

**Background:**

Our study aims to develop a lncRNA-based classifier and a nomogram incorporating the genomic signature and clinicopathologic factors to help to improve the accuracy of recurrence prediction for hepatocellular carcinoma (HCC) patients.

**Methods:**

The lncRNA profiling data of 374 HCC patients and 50 normal healthy controls were downloaded from The Cancer Genome Atlas (TCGA). Using univariable Cox regression and least absolute shrinkage and selection operator (LASSO) analysis, we developed a 15-lncRNA-based classifier and compared our classifier to the existing six-lncRNA signature. Besides, a nomogram incorporating the genomic classifier and clinicopathologic factors was also developed. The predictive accuracy and discriminative ability of the genomic-clinicopathologic nomogram were determined by a concordance index (C-index) and calibration curve and were compared with the TNM staging system by the C-index and receiver operating characteristic (ROC) analysis. Decision curve analysis (DCA) was performed to estimate the clinical value of our nomogram.

**Results:**

Fifteen relapse-free survival (RFS-) related lncRNAs were identified, and the classifier, consisting of the identified 15 lncRNAs, could effectively classify patients into the high-risk and low-risk subgroups. The prediction accuracy of the 15-lncRNA-based classifier for predicting 2-year and 5-year RFS was 0.791 and 0.834 in the training set and 0.684 and 0.747 in the validation set, respectively, which was better than the existing six-lncRNA signature. Moreover, the AUC of genomic-clinicopathologic nomogram in predicting RFS were 0.837 in the training set and 0.753 in the validation set, and the C-index of the genomic-clinicopathologic nomogram was 0.78 (0.72-0.83) in the training set and 0.71 (0.65-0.76) in the validation set, which was better than the traditional TNM stage and 15-lncRNA-based classifier. The decision curve analysis further demonstrated that our nomogram had a larger net benefit than the TNM stage and 15-lncRNA-based classifier. The results were confirmed externally.

**Conclusion:**

Compared to the TNM stage, the 15-lncRNAs-based classifier-clinicopathologic nomogram is a more effective and valuable tool to identify HCC recurrence and may aid in clinical decision-making.

## 1. Introduction

Hepatocellular carcinoma (HCC) is one of the most malignant cancers which represents the fourth leading cause of cancer-related death and the most common cause of mortality of cirrhotic patients. Every year, there are 841,000 patients developed HCC and 782,000 HCC patients died [1]. Till now, only two therapeutic treatments, including surgical resection and liver transplantation, are recommended as the first-line therapy to potentially cure HCC [2]. However, surgical resection is hampered as more than 70% of HCC patients experience disease recurrence approximately at 5 years after resection [3], and donor organ shortages render the large-scale application of liver transplantation. Therefore, identifying reliable and accurate predictive markers/models to screen out which subset of patients with HCC is vulnerable to develop recurrence is urgently needed.

Long noncoding RNAs (lncRNAs) are newly discovered RNA transcripts which were found to play an important role in cell differentiation and development by transcriptionally or posttranscriptionally regulating biological processes [4, 5, 6]. Besides, an increasing number of studies have reported the association of lncRNAs with the development and progression of cancers, including HCC [7, 8]. These studies suggested that lncRNAs may be developed as potentially useful biomarkers in the diagnosis and prognosis of HCC. For example, overexpression of lncRNA LOC90784, RUSC1-AS-N, and RNA AWPPH has been associated with poor clinical features and poorer overall survival in HCC patients [9, 10, 11]. Moreover, a prognostic signature based on lncRNAs has also been found to improve the prognosis prediction of HCC [12, 13], but the predictive value of lncRNA-based signature in the recurrence of HCC remains poorly evaluated.

In the present study, we aimed to develop a lncRNA-based classifier and a nomogram incorporating the genomic signature and clinicopathologic factors to help improve the accuracy of recurrence for HCC patients after surgery. We identified lncRNAs that were significantly associated with relapse-free survival (RFS) of HCC patients from The Cancer Genome Atlas (TCGA) and then used them to construct a lncRNA-based classifier in the training set. A nomogram incorporating the lncRNA-based classifier and clinicopathologic factors was also developed for predicting RFS. Finally, the predictive ability of the nomogram was evaluated and validated in an internal and external validation set.

## 2. Materials and Methods

### 2.1. Ethics Statement

All the data was obtained from TCGA and GEO, and the informed consent had been attained from the patients before our study.

### 2.2. Collection of lncRNA Data and Clinical Characteristics of HCC Patients from TCGA

The lncRNA profiling data of 374 HCC patients and 50 normal healthy controls were downloaded from TCGA. Then, clinical parameters, such as age, gender, family history, alcohol consumption, mutation count, fraction genome altered, BMI, APF, platelet, albumin, creatinine, cirrhosis, histologic grade, T stage, TNM stage, Eastern Cooperative Oncology Group (ECOG), and RFS time, were also downloaded from TCGA. Eighty-one HCC patients were excluded due to RFS time < 1 month or the unavailability of lncRNA data. So, 293 HCC patients with available lncRNA data and clinical characteristics were finally included in our study. Subsequently, 293 HCC patients were randomly assigned to a training set (*N* = 147) and a validation set (*N* = 146) by the R software. Moreover, the GSE76427 dataset (115 HNC tissue samples, 52 adjacent nontumor tissue samples, and 108 tumor samples had complete information of recurrence status and recurrence-free survival time information) from the Gene Expression Omnibus(GEO) (https://www.ncbi.nlm.nih.gov/geo/) was used for external validation.

### 2.3. Construction and Validation of lncRNA-Based Classifier for RFS

**Figure 1:**
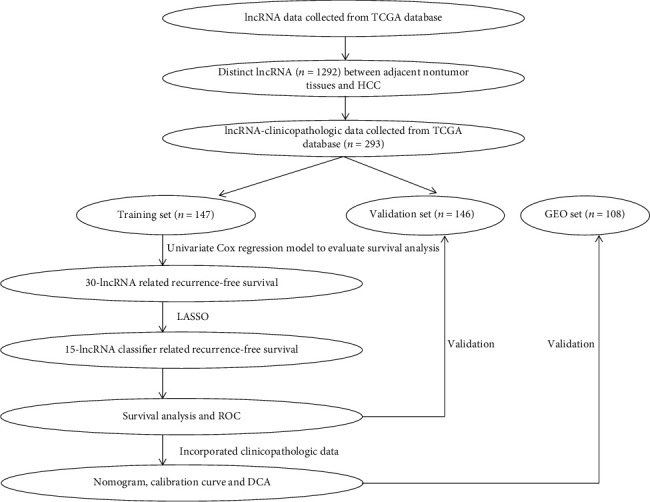
The flowchart of study design. LASSO: least absolute shrinkage and selection operator.

First, the moderated *t*-statistics method and the Benjamini–Hochberg procedure were used to identify distinct differential lncRNAs between normal tissues and HCC tissues. The cutoff criteria of distinct differential lncRNAs were *P* < 0.05 and the false discovery rate (FDR) < 0.05. Next, the univariable Cox regression analysis was used to select RFS-related lncRNAs in the training set. The least absolute shrinkage and selection operator (LASSO) analysis was used to further narrow down the RFS-related lncRNAs, and a signature consisting these well-selected lncRNAs was developed [14]. The LASSO analysis is a popular estimation procedure in multiple linear regression when an underlying design has a sparse structure, as it could set some regression coefficients exactly equal to 0. In our study, we developed a perturbation bootstrap method and established its validity in approximating the distribution of the LASSO in heteroscedastic linear regression. The underlying covariates were allowed to be either random or nonrandom, and the proposed bootstrap method was proved to work irrespective of the nature of the covariates. The simulation study also justified our method in finite samples. In order to obtain the accurate estimate stability of our model, cross-validation was performed, as it could provide unbiased estimation [15]. In the present study, a 5-fold cross-validation was carried out; the 293 HCC patients were divided into 5 subsets of equal size and trained for 5 times, each time leaving out subsets as validation data. The accuracy were 90.3%, 91.1%, 90.8%, 90.4%, and 91%, respectively, indicating the good stability of our 15-lncRNA-based classifier. By this classifier, we calculated the risk scores of HCC patients and then divided patients into the high-risk patients and low-risk subgroup based on the best cutoff value, which was a point when the Youden index (sensitivity + specificity − 1) reached the maximum value in the training set. The RSF difference between the high-risk patients and low-risk patients was further compared by the Kaplan-Meier analysis. The log-rank test was used to compare subgroups. The flowchart of the present study is shown in Figure 1.

### 2.4. Receiver Operating Characteristic (ROC)

To further evaluate the predictive accuracy of the lncRNA-based classifier, an ROC analysis was performed in the training set and validation set. We calculated the area under the ROC curve (AUC) of the lncRNA-based classifier for predicting the 2-year and 5-year RFS and compared the predictive ability of our lncRNA-based signature with other published lncRNA signature [16] for RFS which also developed from the HCC patients of TCGA.

### 2.5. Genomic-Clinicopathologic Nomogram

In order to make the lncRNA-based classifier to be more applicable for clinicians, a lncRNA-based classifier-related nomogram was constructed. First, univariate and multivariate Cox regression analyses were used to identify the clinical risk parameters associated with RFS in the training set. Next, the lncRNA-based classifier, together with the risk parameters, was used to develop a genomic-clinicopathologic nomogram in the training set.

Model performance was evaluated by determining the calibration and discrimination. Discrimination is the model's ability to differentiate between patients who recur from HCC and patients who will not. Discrimination was calculated through the concordance index (C-index). We also illustrated discrimination by dividing the dataset into three groups based on the score generated by the nomogram. We plotted a Kaplan–Meier curve for all three groups.

Calibration of the nomogram was assessed by plotting the observed RFS rate (the mean Kaplan-Meier estimate for patients in each octile) against the nomogram 2- and 5-year predicted RFS probability (i.e., the mean nomogram predicted probability for patients in each octile). A perfectly accurate nomogram prediction model would result in a plot in which the observed and predicted probabilities for the given groups would fall along the 45-degree line. The distance between the pairs and the 45-degree line was a measure of the absolute error of the nomogram's prediction.

The ROC analysis was used to evaluate and compare the discrimination ability of the nomogram with lncRNA-based classifier and TNM stage. Then, the decision curve analysis (DCA) was performed to evaluate the clinical usefulness of the genomic-clinicopathologic nomogram [17, 18]. DCA was performed by calculating the net benefit for a range of threshold probabilities, which place benefits and harms on the same scale. This analysis determined whether clinical decision-making based on a model would do more good than harm. DCA provided straightforward information about the clinical value of a model, in contrast to traditional measures such as sensitivity or specificity, which were abstract statistical concepts.

### 2.6. Gene Ontology (GO) and Kyoto Encyclopedia of Genes and Genomes (KEGG) Analyses of the 15-lncRNA-Based Classifier

To explore the biological function and pathways of the 15-lncRNA-based classifier, GO and KEGG analyses were conducted. First, the Pearson correlation algorithm was performed between these 15 lncRNAs and the protein-coding genes (mRNAs), and the correlation coefficient > 0.4, *P* < 0.001 was considered as significant correlation. Then, the potential biological processes of these lncRNA target genes were further investigated by the Gene Ontology (GO) and Kyoto Encyclopedia of Genes and Genomes (KEGG) analyses in DAVID, a common bioinformatics tool (http://david.abcc.ncifcrf.gov/, version 6.8) [19].

### 2.7. Statistical Analysis

The SPSS statistics 22.0 and R software (R version 3.5.2) were used to conduct the statistical analysis. Univariate and multivariate Cox regression analysis was performed to identify potential predictors associated with RFS. If there were missed data in some of the potential predictors, these missing data would be imputed, as full case analysis would improve the statistical power and reduce potentially biased results. Multiple imputation was used to imput the missing data as the missing data were considered missing at random after analyzing the patterns of them. Multiple imputation was performed MI with the Markov Chain Monte Carlo function, and 5 iterations were used to account for possible simulation errors.

The LASSO analysis was performed with the “glmnet” packages, cross-validation was conducted with the “caret” packages, and ROC analysis was done with the “survivalROC” packages. The nomogram and calibration plots were generated with the “rms” packages, and DCA was performed with the “stdca.R.” A two-sided *P* < 0.05 would be recognized as statistically significant.

## 3. Results

### 3.1. Demographic Parameters and RFS Outcome of HCC Patients

In the present study, 293 HCC patients with available lncRNA data and clinical characteristics were included. The basic clinical characteristics of these HCC patients are summarized in Table 1. The median RFS was 20.99 months (range: 1.22-120.73 months). Of all the 293 HCC patients, 170 (57.9%) patients developed recurrence during follow-up, and the 2-year and 5-year RSF rates were 46.4% and 29.1%, respectively. It was also of note that among the recurrent patients, 143 patients (84.1%) experienced recurrence during the first two years after resection.

### 3.2. Development and Validation of lncRNA-Based Classifier

**Figure 2:**
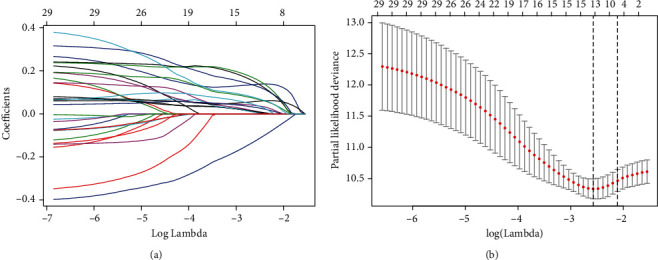
Selection of RFS-associated lncRNAs by the LASSO analysis in the training set. (a) LASSO coefficient profiles of the 30 lncRNAs. A vertical line is drawn at the optimal value by the minimum criteria and results in fifteen nonzero coefficients. The fifteen lncRNAs—AC012625.1, AC068481.1, AC092675.1, AC093772.1, AC109779.1, AC118653.1, AC246785.3, AL121985.1, AL121985.1, AP002478.1, ARHGEF7.AS1, GACAT3, LINC00462, LINC01700, and LINC02429, respectively—were selected in the LASSO Cox regression model. (b) The fifteen lncRNAs selected by the LASSO Cox regression analysis. The two dotted vertical lines are drawn at the optimal values by minimum criteria (left) and 1 - s.e. criteria (right).

First, 1292 distinct differential lncRNAs between normal tissues and HCC tissues were got basing on the filter criteria described in the section of Methods (supplementary material 1). Then, the top 30 RFS-related lncRNAs were identified by univariable Cox regression analysis in the training set (supplementary material 2). Next, the LASSO analysis was used to further narrow down the RFS-related lncRNAs (Figure 2), and we finally selected 15 RFS-related lncRNAs, which were AC012625.1, AC068481.1, AC092675.1, AC093772.1, AC109779.1, AC118653.1, AC246785.3, AL121985.1, AL121985.1, AP002478.1, ARHGEF7.AS1, GACAT3, LINC00462, LINC01700, and LINC02429 (Table 2).

**Figure 3:**
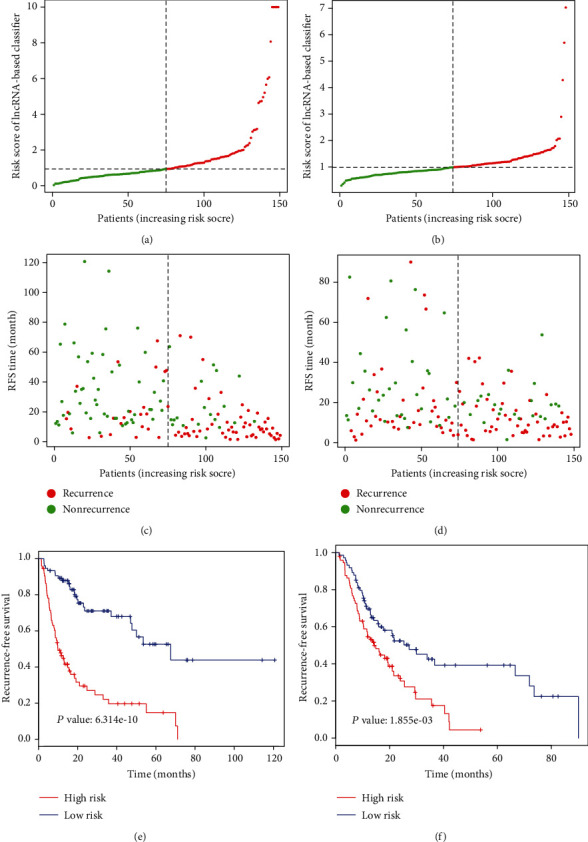
Development and validation of the 15-lncRNAs-based classifier for HCC recurrence. (a), (c) Distribution of lncRNA-based classifier risk score in the training set. (b), (d) Distribution of lncRNA-based classifier risk score in the validation set. (e) The Kaplan-Meier analysis of RFS time for the high-risk and low-risk patients of the training set. (f) The Kaplan-Meier analysis of the RFS time for the high-risk and low-risk patients of the validation set.

On the basis of the coefficients weighted by the LASSO analysis, a classifier was developed, and the risk score was as follows: risk score = (0.05174×AC012625.1) + (0.10870×AC068481.1) + (0.05747×AC092675.1) + (0.05770×AC093772.1) + (-0.32522×AC109779.1) + (0.06050×AC118653.1)+(0.05021×AC246785.3) + (0.03112×AL121985.1) + (0.12532×AL512604.2) + (0.06333×AP002478.1) + (0.10057×ARHGEF7.AS1) + (0.26230×GACAT3) + (0.25478×LINC00462) + (0.21032×LINC01700) + (0.12948×LINC02429). With this classifier, the risk score for every HCC patient would be calculated, and then, they were classified into high-risk patients and low-risk patients according to the best cutoff (described in Materials and Method). As was shown in Figure 3, patients with a high risk score were more likely to develop recurrence and had shorter RFS than those with a low risk score in the training set (9.89 vs. 67.58 months, HR = 3.96, 95% CI: 2.5-6.3, *P* ≤ 0.001). Verification analysis was further performed in the validation set, in the total cohort, and external validation set. Similarly, the 15-lncRNA-based classifier could also classify patients into the high-risk and the low-risk subgroups by the same cutoff value. The median RFS time of the high-risk patients was shorter than that of the low-risk patients in the validation set (14.22 vs. 27.2 months, HR = 1.941, 95% CI: 1.28-2.94, *P* ≤ 0.001), in the total cohort (*P* < 0.001, Figure S1A), and external validation set (*P* ≤ 0.001, Figure S2C). Besides, the 15-lncRNA-based classifier could also classify patients into the high-risk subgroup with shorter overall survival (OS) time and the low-risk subgroup with longer OS time no matter in the training set, the validation set, or the total cohort (all *P* < 0.001, Figure S3A, Figure S3C, Figure S1C). Taken together, these results suggested that the 15-lncRNA-based classifier could effectively classify HCC patients into two distinct subgroups with high risk or low risk of recurrence or OS.

### 3.3. Predictive Value of 15-lncRNA-Based Classifier and Comparison with Other lncRNA-Based Classifier from TCGA

**Figure 4:**
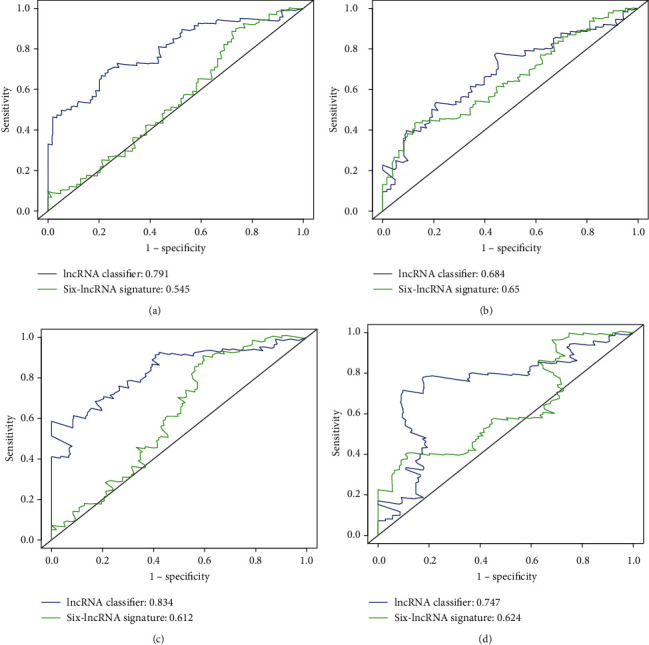
Comparison of the predictive value of the 15-lncRNA-based classifier with other lncRNA-based signature from TCGA. The ROC analysis was performed to assess the predictive value of the 15-lncRNA-based classifier and the 6-lncRNA-based signature. (a) The AUC of 15-lncRNA-based classifier in predicting the 2-year RFS time. (b) The AUC of the 6-lncRNAs-based signature in predicting the 2-year RFS time. (c) The AUC of the 15-lncRNA-based classifier in predicting the 5-year RFS time. (d) The AUC of 6-lncRNA-based signature in predicting the 2-year RFS time.

Recently, a six-lncRNA-based signature was developed and validated by Gu et al. to predict RFS with the data of HCC patients from TCGA [16]. To compare the predictive value of our 15-lncRNA-based classifier with the six-lncRNA-based signature, the ROC curve analysis was performed. As was shown in Figure 4, the AUC of the 15-lncRNA-based classifier for predicting 2-year and 5-year RFS in the training set were 0.791 and 0.834, respectively, while that of the six-lncRNA-based signature were 0.545 and 0.612, respectively (Figures 4(a) and 4(c)). Similar results were also found in the internal validation set and the external validation set. The AUC of the 15-lncRNA-based classifier for predicting the 2-year and 5-year RFS were 0.684 and 0.747 in the internal validation set, respectively, and the AUC of the six-lncRNA-based signature were 0.65 and 0.624, respectively (Figures 4(b) and 4(d)). The AUC of the 15-lncRNA-based classifier for predicting the 2-year and 5-year RFS were 0.743 and 0.651 in the external validation set (Figure S2D). Besides, the 15-lncRNA-based classifier also had a good prognostic value for RFS of the 293 HCC patients (total cohort) as the AUC of the 15-lncRNA-based classifier for predicting the 2-year and 5-year RFS were 0.753 and 0.814, respectively (Figure S1B). What is more, we also examined the prognostic value of the 15-lncRNA-based classifier for OS. The AUC of the 15-lncRNA-based classifier for predicting the 2-year and 5-year OS were 0.846 and 0.816 in the training set (Figure S3B), 0.791 and 0.804 in the validation set (Figure S3D), and 0.828 and 0.805 in the total cohort (Figure S1D). These results suggested that the 15-lncRNA-based classifier demonstrated better performance in predicting RFS than the six-lncRNA-based signature.

### 3.4. Development of Genomic-Clinicopathologic Nomogram

**Figure 5:**
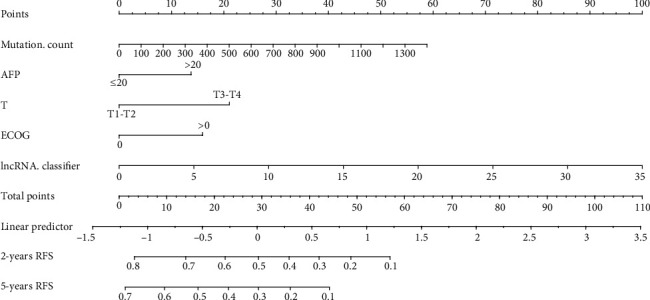
Development of the 15-lncRNAs-based classifier-clinicopathologic nomogram for predicting the 2-year and 5-year RFS.

**Figure 6:**
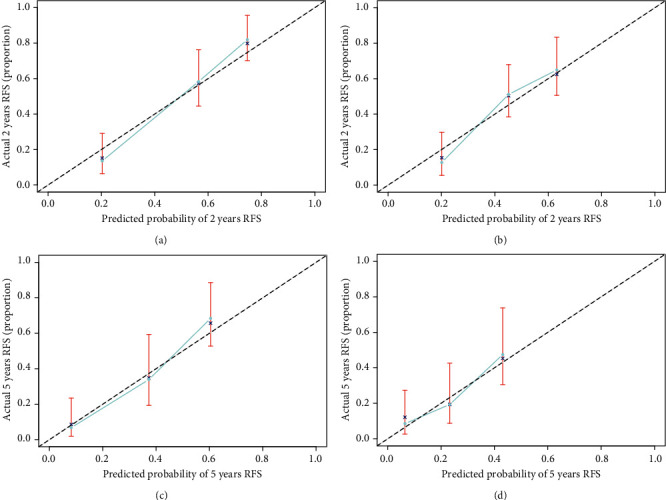
Calibration curve for the 15-lncRNA-based classifier-clinicopathologic nomogram in predicting the 2-year (a) and 5-year (c) RFS time in the training set and the 2-year (b) and 5-year (d) RFS time in the validation set.

To make the lncRNA-based classifier to be more applicable for clinicians, a 15-lncRNA-based classifier-clinicopathologic nomogram was developed to predict the 2-year and 5-year RFS in HCC patients. The potential predictors associated with RFS were identified by univariate and multivariate Cox regression analysis in the training set. The univariable Cox regression analysis showed that mutation count, BMI, APF, liver cirrhosis, tumor stage, TNM stage, ECOG, and the 15-lncRNA-based classifier were related with RFS, and the multivariate Cox regression analysis further showed that mutation count, AFP, T stage, ECOG, and the 15-lncRNA-based classifier were independent predictors of RFS (Table 3). So, these five predictors were used to develop the genomic-clinicopathologic nomogram (Figure 5), which would help clinicians to preoperatively predict the recurrence risk in HCC patients. The C-index of the genomic-clinicopathologic nomogram was 0.78 (0.72-0.83) (Table 4), and the calibration plots exhibited good consistency between the predicted RFS and the actual RFS (Figures 6(a), 6(c)). Likewise, consistent results were also found in the validation set. The C-index of the genomic-clinicopathologic nomogram in the validation set was 0.71 (0.65-0.76) (Table 4) and also showed good consistency between the predicted RFS and the actual RFS (Figures 6(b) and 6(d)). Additionally, the tertiles of all the total points were used to divide the patients into high-, intermediate-, and low-risk groups with distinct RFS time or OS time. The Kaplan-Meier analysis (log-rank *P* ≤ 0.001) of the three risk subgroups indicated the great utility of the genomic-clinicopathologic nomogram in the training set (Figure S4A, Figure S5A), in the validation set (Figure S4B, Figure S5B), and in the total cohort (Figure S6A, S6B). All these results indicated the perfect performance of our genomic-clinicopathologic nomogram.

**Figure 7:**
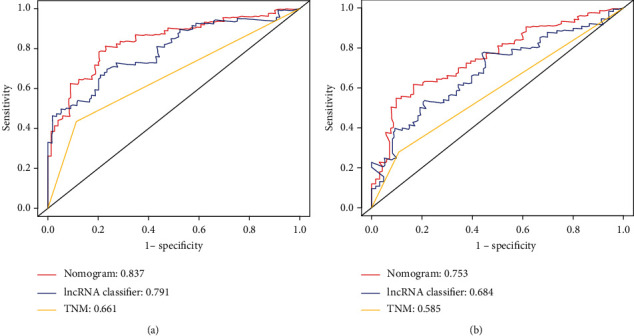
Comparison of the predictive value of the 15-lncRNA-based classifier-clinicopathologic nomogram with the TNM stage and the 15-lncRNA-based classifier. ROC analysis was used to evaluate the predictive accuracy of the three models in predicting the RFS time of the training set (a) and the validation set (b).

To further evaluate the predictive ability of the genomic-clinicopathologic nomogram, we compared the C-index and ROC analysis results of the genomic-clinicopathologic nomogram with the AJCC TNM stage and the 15-lncRNA-based classifier in the training set and validation set. As was shown in Table 4, the C-index of the genomic-clinicopathologic nomogram was higher than that of the TNM stage (0.64 (0.58-0.69) in the training set and 0.56 (0.51-0.60) in the validation set) and the 15-lncRNA-based classifier (0.74 (0.68-0.80) in the training set and 0.61 (0.55-0.68) in the validation set). The likelihood ratio test, the linear trend *χ*^2^ test, and the Akaike information criterion all demonstrated that the genomic-clinicopathologic nomogram had higher prediction efficiency than the TNM stage or 15-lncRNA-based classifier alone. Similar to C-index, the ROC analysis also indicated that the genomic-clinicopathologic nomogram (AUC 0.837 for the training set and 0.753 for the validation set) was better than the TNM stage (AUC 0.661 for the training set and 0.585 for the validation set) or 15-lncRNA-based classifier (AUC 0.791 for the training set and 0.684 for the validation set) alone in predicting RFS (Figures 7(a) and 7(b)).

**Figure 8:**
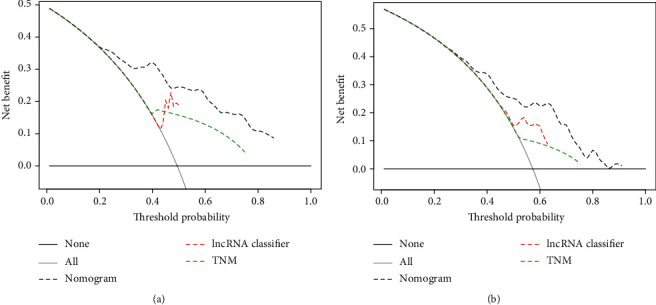
Decision curve analysis (DCA) analysis for the 15-lncRNA-based classifier-clinicopathologic nomogram with TNM stage and 15-lncRNA-based classifier in the training set (a) and the validation set (b).

Finally, the clinical usefulness of the genomic-clinicopathologic nomogram was assessed by the DCA, an abstract statistical concept, which gave visualized information on the clinical value of a model. As were presented in Figures 8(a) and 8(b), the DCA results showed that the HCC recurrence-associated treatment decision based on the genomic-clinicopathologic nomogram resulted in more net benefit than the treatment decision based on the TNM stage or 15-lncRNA-based classifier, or treating either all patients or none in the training set and the validation set.

### 3.5. Biological Function and Pathways of 15-lncRNA-Based Classifier

To explore the biological function and pathways of the 15-lncRNA-based classifier, the GO and KEGG analyses were performed. As is shown in Figure S6, biological processes such as GO:0048037 (cofactor binding), GO:0050662 (coenzyme binding), GO:0016614 (oxidoreductase activity, acting on CH-OH group of donors), GO:0009055 (electron transfer activity), and GO:0016616 (oxidoreductase activity, acting on the CH-OH group of donors, NAD or NADP as acceptor) were mainly regulated by the 15-lncRNA-based classifier (Figure S7A). The KEGG analysis also showed that carbon metabolism, peroxisome, fatty acid degradation, valine, leucine, and PPAR signaling pathway were mainly affected by the 15-lncRNA-based classifier (Figure S7B). These results suggested an important role played by abnormal metabolism in hepatocellular carcinogenesis. Our findings were in accordance with the well-known viewpoint that alterations in cellular metabolism were hallmarks of cancer, and various works suggested that lipid and glucose metabolism took an active role in hepatocellular carcinogenesis [20], which provided evidence for the rationality and molecular thesis of the 15-lncRNA-based classifier.

## 4. Discussion

Basing on the lncRNA profiling data and clinical parameter of 293 HCC patients from TCGA, we identified 15 lncRNAs related to RFS. On the basis of these lncRNAs, we developed and validated a classifier, which could effectively classify patients into high-risk patients with shorter RFS and low-risk patients with longer RFS. The 15-lncRNA-based classifier is significantly associated with tumor recurrence and provides accurate prediction in predicting the 2-year and 5-year RFS of HCC patients. More importantly, we also construct and validate the lncRNAs–clinicopathologic nomogram incorporating the clinicopathologic parameter of mutation count, AFP, T stage, and ECOG, which might contribute to the individual evaluation of RFS in HCC patients after surgical resection.

An increasing number of studies have found that lncRNAs may be exploited as potential effective biomarkers in the diagnosis and prognosis of HCC [9, 11]. Yan et al. reported that seven lncRNAs, including AC009005.2, RP11-363N22.3, RP11-932O9.10, RP11-572O6.1, RP11-190C22.8, RP11-388C12.8, and ZFPM2-AS1, were associated with OS of HCC patients, and the seven-lncRNA signature could also divide patients into the high-risk and low-risk groups with significantly different OS [13]. Wu et al. found that MIR22HG, CTC-297N7.9, CTD-2139B15.2, RP11-589N15.2, RP11-343N15.5, and RP11-479G22.8 were independent predictors of HCC patients' OS, and a signature consisted of these six lncRNAs can effectively classify patients into high-risk patients with shorter survival time and low-risk patients with longer survival, and the six-lncRNA signature exhibited superior predictive capacity than the TNM stage [12]. These studies suggested the potential clinical implications of lncRNA-based signature in improving the prognosis prediction of HCC. However, it should be noted that OS is more likely to be influenced by postrecurrence treatment and liver function and RFS could more accurately reflect the biologic behavior for HCC; thus, in the present study, we tried to identify the RFS-related lncRNAs and developed and validated a classifier, which may be more valuable for HCC patient management.

Recently, Gu et al. develop and validate a six-lncRNA-based signature to predict RFS also with the data of HCC patients from TCGA [16]. They demonstrated that MSC-AS1, POLR2J4, EIF3J-AS1, SERHL, RMST, and PVT1 were significantly upregulated in tumor samples compared to nontumor samples and were significantly associated with RFS. Different from the abovementioned six lncRNAs, we identified another 15 RFS-related lncRNAs, including AC012625.1, AC068481.1, AC092675.1, AC093772.1, AC109779.1, AC118653.1, AC246785.3, AL121985.1, AL121985.1, AP002478.1, ARHGEF7.AS1, GACAT3, LINC00462, LINC01700, and LINC02429 in the present study. The reason for the differences between our findings and other studies may be attributable to the difference in training datasets. TCGA dataset was used as the training dataset in the present study, while GSE76427 was used in the study by Gu et al. The training dataset may finally determine the key RFS-lncRNAs for further investigation. However, it should be noted that the performance of a classifier lay on its prediction sensitivity and specificity. The prediction accuracy of the 15-lncRNA-based classifier for predicting 2-year and 5-year RFS were 0.791 and 0.834 in the training set and 0.684 and 0.747 in the validation set, while the AUC of the 6-lncRNA-based signature were 0.545 and 0.612 in the training set and 0.65 and 0.624 in the validation set, suggesting that our 15-lncRNA-based classifier exhibited better efficiency in predicting RFS.

Except for the six-lncRNA-based signature, Gu et al. also developed another lncRNA-based signature for HCC recurrence in patients with small HCC (maximum tumor diameter ≤ 5 cm) [21]. In his study, a 3-lncRNA-based signature, which consists of LOC101927051, LINC00667, and NSUN5P2, was developed and validated for predicting OS and RFS in patients with small HCC. This 3-lncRNA-based signature was more suitable for patients with higher serum levels of AFP (>20 ng/mL) and relatively lower levels of albumin (<4.0 g/dL) or Asian patients with no family history of HCC or history of alcohol consumption. Similar to our study, Gu et al. also constructed a lncRNAs–clinicopathologic nomogram incorporating clinicopathologic parameter of liver cirrhosis and ECOG for predicting the 1-year and 3-year RFS with a C-index of 0.633. Compared to the 3-lncRNA-based clinicopathologic nomogram, our 15-lncRNAs–clinicopathologic nomogram demonstrated better predictive ability (C-index of 0.78 in the training set and 0.71 in the validation set). Additionally, the nomogram by Gu et al. was suitable for patients with small HCC, while our nomogram was developed for predicting the recurrence of all HCC patients, potentially contributing to the prediction of recurrence in broader patients with HCC. Secondly, the nomogram by Gu et al. was used for predicting the 1-year and 3-year RFS, while our nomogram was developed for predicting the 2-year and 5-year RFS, which was more fit with early recurrence (recurrence within 2 years) and long-term recurrence (recurrence within 5 years). Finally, we also validated our nomogram in an internal validation set to validate the stability of our nomogram, which was not performed in the study by Gu et al. Moreover, our nomogram appears to be a favorable predictive model compared to the TNM stage with a C-index of 0.64 in the training set and 0.56 in the validation set, indicating that our genomic-clinicopathologic nomogram may represent a reliable and accurate model for HCC recurrence prediction, which is helpful for individualized therapeutic treatment decision and posttreatment follow-up decision-making.

Consistent with previous studies, mutation count, AFP, T stage, and ECOG were found to be significantly associated with HCC recurrence in the present study [21, 22, 23, 24]. In addition to these clinical factors, as expected, the 15-lncRNA-based signature was also found to be significantly related to HCC recurrence in our study. Till now, only a few validated clinical nomograms for HCC recurrence have been reported [25, 26, 27]. For example, a nomogram consisting of 7 clinical factors, including age, AFP, PT, magnitude of hepatectomy, postoperative complication, number of tumor nodules, and microvascular invasion, was developed and validated using the data of 617 HCC patients. However, the results limited its use for HCC patients beyond the Milan criteria [26]. Another nomogram incorporating sex, the log of calculated tumor volume, ALB, platelet count, and microvascular invasion was well-constructed and validated by Shim et al. with data from 1085 HCC patients. However, this model could only be applied for early-stage HCC patients, and the authors only evaluated the prediction accuracy for predicting the 2-year RFS [27]. Different from the nomogram described above, we developed and validated a nomogram incorporating the clinicopathologic parameter and genomic data, which may help to improve the stability and accuracy of the prediction probability of the nomogram [28].

Among the 15 RFS-related lncRNAs, AP002478.1, GACAT3, and LINC00462 have been previously reported to be related to cancers. AP002478.1 has been reported to be potential prognostic biomarkers for HCC patients and gastric cancer patients [29, 30]. GACAT3 is the first to be found to significantly overexpress in gastric cancer tissues and gastric cancer cell line MGC-803. The higher expression of GACAT3 significantly was associated with tumor size, distant metastasis, TNM stages, and shorter OS [31, 32]. One mechanism study showed that GACAT3 knockdown could significantly inhibit proliferation, colony formation, migration, and invasion of GC cells by regulating miR-497, while the downregulation of GACAT3 decreased its tumorigenesis [33]. Moreover, GACAT3 was also found to similar tumorigenesis in breast cancer, glioma, and colorectal cancer [34, 35, 36]. The upregulation of LINC00462 was found to be associated with larger tumor size, poorer tumor differentiation, TNM stage, and metastasis of pancreatic cancer patients. Mechanism study indicated that LINC00462 could promoted proliferation, migration invasion of pancreatic cancer cells by regulating miR-665 [37]. Notably, LINC00462 was also significantly overexpressed in HCC tissues, and knockdown of LINC00462 inhibited the aggressive oncogenic phenotype in HCC cells by regulating PI3K/AKT the signaling pathway, suggesting LINC00462 may be a potential and promising therapeutic target for HCC [38]. Therefore, further research on the biological function of these identified lncRNAs may shed light on the HCC recurrence.

Although our genomic-clinicopathologic nomogram demonstrated impressive performance in the HCC recurrence prediction, the limitation of this study should also be noted. First, our nomogram is limited by the retrospective collection of data and fails to include some already recognized RFS-related factors (e.g., liver cirrhosis, vascular invasion) and some important molecular factors (e.g., TP53 mutation). Further efforts to incorporate more geographic and molecular factors will potentially help to improve the performance of the present model. Second, there is no eternal or prospective validation for the genomic-clinicopathologic nomogram in the present study, so external and multicenter prospective cohorts with large sample sizes are still needed to validate the clinical application of our model. Finally, we do not explore the underlying biological function and pathways of the genomic classifier, so further mechanism studies are needed to uncover the related mechanisms.

In conclusion, we develop a lncRNAs–clinicopathologic nomogram and demonstrate that it appears to be a more effective tool for HCC recurrence prediction, compared to the TNM stage and other lncRNA-based signature from TCGA. The lncRNAs–clinicopathologic nomogram may help clinicians to make more fitly individualized therapeutic strategies for HCC patients.

## Figures and Tables

**Table 1:** Characteristics of the patients in the training set and validation set.

Variable	Category	Training set		Validation set	
		(*n* = 147)	%	(*n* = 146)	%
Age (years)	Median	60		62	
	Range(years)	18-82		25-90	
	NA	0	0	0	0
Sex	Male	101	68.7	103	70.5
	NA	0	0	0	0
Race	Asian	69	46.9	63	43.2
	White	72	49.0	70	47.9
	Black or African American	4	2.7	8	5.5
	NA	2	1.4	5	3.4
Family history	Yes	44	29.9	45	30.8
	NA	22	15.0	18	12.3
HBV	Yes	49	33.3	44	30.1
	NA	7	4.8	7	4.8
HCV	Yes	19	12.9	23	15.8
	NA	7	4.8	7	4.8
Alcohol consumption	Yes	46	31.3	55	37.7
	NA	7	4.8	7	4.8
Mutation count	Median	79.5		83	
	Range	12-1323		1-685	
	NA	7	4.8	2	1.4
Fraction genome altered	Median	0.25		0.24	
	Range	0-0.85	0	0-0.93	
BMI	NA	3	2.0	2	1.4
	≤24	60	40.8		
	>24	74	50.3		
	NA	13	8.8		
AFP (ng/mL)	≤20	59	40.1	71	48.6
	>20	59	40.1	42	28.8
	NA	29	19.7	33	22.6
Platelet (×109/L)	≤200	57	38.8	56	38.4
	>200	74	50.3	65	44.5
	NA	16	10.9	25	17.1
Albumin (g/dL)	≤4.0	62	42.2	60	41.1
	>4.0	63	42.9	61	41.8
	NA	22	15.0	25	17.1
Creatinine (mg/dL)	<1.1	100	68	91	62.3
	≥1.1	26	17.7	27	18.5
	NA	21	14.3	28	19.2
Liver cirrhosis	Yes	51	34.7	67	45.9
	NA	64	43.5	53	36.3
Histological grade	G1-G2	83	56.5	97	66.4
	G3-G4	62	42.2	47	32.2
	NA	2	1.4	2	1.4
T stage	T1-T2	105	71.4	115	78.8
	T3-T4	41	27.9	30	20.5
	NA	1	0.7	1	0.7
TNM stage	StageI-II	101	68.7	105	71.9
	StageIII-IV	39	26.5	30	20.5
	NA	7	4.8	11	7.5
ECOG	0	79	53.7	68	46.6
	>0	44	29.9	54	37.0
	NA	24	16.3	24	16.4

Abbreviations: NA: not available; AFP: alpha-fetoprotein.

**Table 2:** Multivariable Cox regression analysis of lncRNA with recurrence-free survival in the training set.

lncRNA	Coef	Exp (coef)	Lower.95	Upper.95	*P* value
AC012625.1	0.05174	1.0531	0.8952	1.2389	0.5325
AC068481.1	0.10870	1.1148	0.8413	1.4773	0.4492
AC092675.1	0.05747	1.0592	0.8497	1.3202	0.6092
AC093772.1	0.05770	1.0594	0.8869	1.2654	0.5245
AC109779.1	-0.32522	0.7224	0.5408	0.9649	0.0277^∗^
AC118653.1	0.06050	1.0624	0.7956	1.4187	0.6818
AC246785.3	0.05021	1.0515	0.8937	1.2371	0.5449
AL121985.1	0.03112	1.0316	0.9067	1.1737	0.6364
AL512604.2	0.12532	1.1335	0.9463	1.3577	0.1736
AP002478.1	0.06333	1.0654	0.9115	1.2452	0.4262
ARHGEF7.AS1	0.10057	1.1058	0.9219	1.3264	0.2786
GACAT3	0.26230	1.2999	1.0439	1.6187	0.0191^∗^
LINC00462	0.25478	1.2902	1.0717	1.5531	0.0071^∗∗^
LINC01700	0.21032	1.2341	0.9076	1.6779	0.1797
LINC02429	0.12948	1.1382	0.8675	1.4935	0.3503

Signif. codes: 0 “^∗∗∗^”, 0.001 “^∗∗^” 0.01 “^∗^”, 0.05 “.”, and 0.1 “ ” 1.

**Table 3:** Univariable and multivariable Cox regression analysis for prediction of RFS.

Factors	Subgroup	Univariable analysis		Multivariable analysis	
		HR (95% CI)	*P*	HR (95% CI)	*P*
Age		1.00 (0.99-1.02)	0.673	NA	NA
Sex	Female	1			
	Male	0.89 (0.57-1.39)	0.621	NA	NA
Race	Asian	1			
	White	1.15 (0.75-1.78)	0.522	NA	NA
	Black or African American	0.96 (0.34-2.70)	0.932	NA	NA
Family history	No	1			
	Yes	0.68 (0.42-1.09)	0.109	NA	NA
HBV	No	1			
	Yes	0.80 (0.51-1.28)	0.359	NA	NA
HCV	No	1			
	Yes	1.64 (0.98-2.64)	0.058	NA	NA
Alcohol consumption	No	1			
	Yes	1.04 (0.67-1.62)	0.863	NA	NA
Mutation count		1.02 (1.01-1.03)	0.007^∗^	1.02 (1.00-1.03)	0.032^∗^
Fraction genome altered		2.86 (0.97-8.42)	0.057	NA	NA
BMI	≤24	1			
	>24	0.57 (0.37-0.89)	0.014^∗^	0.76 (0.47-1.04)	0.084
AFP	≤20	1		1	
	>20	1.79 (1.14-2.82)	0.012^∗^	1.82 (1.14-2.90)	0.012^∗^
Platelet	≤200	1			
	>200	1.26 (0.79-2.00)	0.328	NA	NA
Albumin	≤4.0	1			
	>4.0	0.96 (0.60-1.52)	0.855	NA	NA
Creatinine	<1.1	1			
	≥1.1	0.81 (0.47-1.40)	0.445	NA	NA
Liver cirrhosis	No	1			
	Yes	2.17 (1.14-4.11)	0.018^∗^	1.56 (0.87-2.12)	0.122
Histological grade	G1-G2	1			
	G3-G4	1.07 (0.68-1.67)	0.772	NA	NA
T stage	T1-T2	1			
	T3-T4	3.12 (1.98-4.92)	*≤*0.001^∗^	2.50 (1.54-4.05)	*≤*0.001^∗^
TNM stage	Stage I-II	1			
	Stage III-IV	2.71 (1.71-4.30)	*≤*0.001^∗^	1.12 (0.47-2.69)	0.801
ECOG	0	1			
	>0	2.41 (1.54-3.77)	*≤*0.001^∗^	2.00 (1.23-3.23)	0.005^∗^
lncRNA classifier		1.13 (1.09-1.18)	*≤*0.001^∗^	1.13 (1.09-1.18)	*≤*0.001^∗^

Abbreviations: HR: hazard ratio; CI: confidence intervals; NA: not available. These variables were eliminated in the multivariate Cox regression model, so the HR and *P* values were not available. ^∗^*P* < 0.05.

**Table 4:** Assessing the prognostic performance of the AJCC stage and nomogram in the training set and validation set.

Cohort	Model	Homogeneity monotonicity and discriminatory ability			Akaike information criterion (AIC)^∗∗∗∗^
		Likelihood ratio (LR) test^∗^	Linear trend *χ*^2^ test^∗∗^	C-index (95% CI)^∗∗∗^	
Training set	TNM stage	16.4	19.6	0.64 (0.58-0.69)	663
	Nine lncRNA classifier	25.6	82.7	0.74 (0.68-0.80)	654
	Nomogram	62.7	112.1	0.78 (0.72-0.83)	625
Validation set	TNM stage	5.8	6.6	0.56 (0.51-0.60)	765
	Nine lncRNA classifier	12.2	21.4	0.61 (0.55-0.68)	758
	Nomogram	41.6	59.2	0.71(0.65-0.76)	737

^∗^The higher homogeneity likelihood ratio indicates a smaller difference within the staging system; it means better homogeneity. ^∗∗^The higher discriminatory ability linear trend indicates a higher linear trend between the staging system; it means better discriminatory ability and gradient monotonicity. ^∗∗∗^A higher C-index means better discriminatory ability. ^∗∗∗∗^Smaller AIC values indicate better optimistic prognostic stratification.

## Data Availability

The data that support the findings of this study are provided in supplementary materials and are also made available in the TCGA via the Genomics Data Commons https://gdc.cancer.gov/.

## Abbreviations

HCC: Hepatocellular carcinoma
lncRNAs: Long noncoding RNAs
OS: Overall survival
RFS: Relapse-free survival
TCGA: The Cancer Genome Atlas
ECGO: Eastern Cooperative Oncology Group
FDR: False discovery rate
ROC: Receiver operating characteristic
LASSO: Least absolute shrinkage and selection operator
AUC: Area under curve
DCA: Decision curve analysis.

## Supplementary Materials

Supplementary 1 Based on the primary filter criteria mentioned in the methods section, we obtained a list of 1160 different lncRNAs.

Supplementary 2 Using univariable Cox regression analysis, we identified 31 prognostic related lncRNAs.
